# Simulation and comparative analysis of binding modes of nucleoside and non-nucleoside agonists at the A_2B_ adenosine receptor

**DOI:** 10.1186/2193-9616-1-24

**Published:** 2013-12-20

**Authors:** Diego Dal Ben, Michela Buccioni, Catia Lambertucci, Ajiroghene Thomas, Rosaria Volpini

**Affiliations:** School of Pharmacy, Medicinal Chemistry Unit, University of Camerino, via S. Agostino 1, Camerino, MC 62032 Italy

**Keywords:** Purinergic receptors, Adenosine receptors, Adenosine receptor agonists, Nucleosides, Purine derivatives, Pyridine derivatives, Molecular modelling, Homology modelling, Molecular docking

## Abstract

**Purpose:**

A_2B_ receptor agonists are studied as possible therapeutic tools for a variety of pathological conditions. Unfortunately, medicinal chemistry efforts have led to the development of a limited number of potent agonists of this receptor, in most cases with a low or no selectivity versus the other adenosine receptor subtypes. Among the developed molecules, two structural families of compounds have been identified based on nucleoside and non-nucleoside (pyridine) scaffolds. The aim of this work is to analyse the binding mode of these molecules at 3D models of the human A_2B_ receptor to identify possible common interaction features and the key receptor residues involved in ligand interaction.

**Methods:**

The A_2B_ receptor models are built by using two recently published crystal structures of the human A_2A_ receptor in complex with two different agonists. The developed models are used as targets for molecular docking studies of nucleoside and non-nucleoside agonists. The generated docking conformations are subjected to energy minimization and rescoring by using three different scoring functions. Further analysis of top-score conformations are performed with a tool evaluating the interaction energy between the ligand and the binding site residues.

**Results:**

Results suggest a set of common interaction points between the two structural families of agonists and the receptor binding site, as evidenced by the superimposition of docking conformations and by analysis of interaction energy with the receptor residues.

**Conclusions:**

The obtained results show that there is a conserved pattern of interaction between the A_2B_ receptor and its agonists. These information and can provide useful data to support the design and the development of A_2B_ receptor agonists belonging to nucleoside or non-nucleoside structural families.

**Electronic supplementary material:**

The online version of this article (doi:10.1186/2193-9616-1-24) contains supplementary material, which is available to authorized users.

## Background

**Figure 1:**
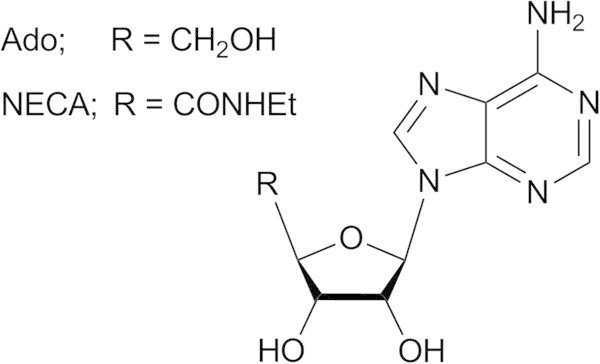
**Molecular structure of Ado and NECA.**

Adenosine (Ado, Figure 1) is a naturally occurring nucleoside that mediates numerous physiological and pathological processes with effects on heart rate and atrial contractility, vascular smooth muscle tone, release of neurotransmitters, lipolysis, as well as renal, platelet, and white blood cell functions (Cristalli and Volpini 2003). Ado activity is mediated by the activation of four receptors that have been cloned (Robeva et al. 1996) and classified as A_1_, A_2A_, A_2B_, and A_3_ adenosine receptor (AR) subtypes (Fredholm et al. 2001) by considering the respective coupling to second messengers. Furthermore, ARs can be distinguished on the basis of their tissue distribution and unique pharmacological profiles. These membrane proteins, belonging to the G protein-coupled receptor (GPCR) family, are hot targets due to their therapeutic potential even though the lack of potent and selective ligands for all subtypes still represents a weakness for the attribution of specific biological activity and for the development of therapeutic tools.

Among ARs, the A_2B_AR has been usually defined “low-affinity AR” due to its lower affinity for Ado and for other agonists respect to the other AR subtypes (Feoktistov and Biaggioni 1997; Beukers et al. 2000). Its stimulation leads to activation of phospholipase C and adenylyl cyclase through the coupling to the Gq and Gs proteins, respectively. The A_2B_AR is widely expressed in the human body and regulates several biological events at cardiovascular, muscular, and central nervous systems, and also in cell growth and during inflammation (Feoktistov and Biaggioni 1997). Hence, this receptor is of therapeutic interest for its targeting in several conditions (Baraldi et al. 2009). In particular, the agonists of this receptor have been evaluated for their cardioprotective effect, due to their reduced bradycardic and hypotensive side effects respect to Ado (Kuno et al. 2007; Philipp et al. 2006; Gao and Jacobson 2007; Eckle et al. 2007), for the treatment of coronary artery disease (Hinschen et al. 2003; Ansari et al. 2007; Kemp and Cocks 1999), and to promote angiogenesis (Feoktistov et al. 2003; Feoktistov et al. 2004). Further applications of A_2B_AR agonists have been explored on the basis of the anti-inflammatory effect following the activation of this receptor, leading to the suggestion of the use of these molecules in septic shock (Kreckler et al. 2006). Even the use of A_2B_AR agonists for the treatment of renal diseases, hypertension, cystic fibrosis, diabetes, and pulmonary diseases associated with hyperplasia has been considered (Volpini et al. 2003; Dubey et al. 2005).

Many efforts of medicinal chemistry have been focused to the development of potent and selective AR agonists and antagonists. In the case of the A_2B_AR, for several years the nucleoside 4’-*N*-ethylcarboxamidoAdo (NECA, Figure 1, EC_50_ = 140 nM) has represented and is still used as reference agonist of this AR subtype, despite its lack of subtype selectivity. Further agonists have been developed by modifying the Ado and NECA structures with the insertion of substituents at the 2- and *N*^6^-positions. The *N*^6^-substituents contain in most cases substituted aromatic rings and polar groups directly bound to 6-amine or to the aromatic function itself. A series of NECA derivatives was developed by inserting at the *N*^6^-position a large substituent similar to the one present at the 8-position of xanthine derivatives that behave as potent A_2B_AR antagonists. Table 1 presents some examples of nucleosides presenting agonist activity at the A_2B_AR. The activity data are taken from a review article by Baraldi and co-workers (Baraldi et al. 2009). In general, the NECA derivatives present higher potency respect to the corresponding Ado derivatives. In any case, while the obtained potencies at the A_2B_AR are at nanomolar level, the selectivity versus the other AR subtypes is still low (Baraldi et al. 2009; Volpini et al. 2002; Lambertucci et al. 2003; Gao et al. 2004; Baraldi et al. 2007a; Baraldi et al. 2007b).

**Table 1:** **Nucleoside A**_**2B**_ **AR agonists analysed in this work (see Additional file** **1** **for structural details)**

cpd	A_1_AR***K***_i_, nM	A_2A_AR***K***_i_, nM	A_2B_AR EC_50_, nM	A_3_AR***K***_i_, nM
**1**	0.67	1.8	920	1.4
**2**	221	9.3	3490	54.2
**3**	1050	1550	82	> 5000
**4**	2600	4100	175	> 5000
**5**	30.5	> 1000	42.6	107
**6**	2.1	2.0	220	0.75

The activity data are taken from Baraldi and co-workers (Baraldi et al. 2009).

A structural novelty on the development of A_2B_AR agonists has been provided by the publication of a series of pyridine derivatives presenting low nanomolar potency at this AR subtype and in some cases remarkable selectivity versus the other ARs. These compounds present two cyano groups at the 3- and 5-positions, an amino function at the 6-position, and two substituents at the remaining 2- and 4-positions presenting various profiles. In general, the 4-substituent contains an aromatic group with possible substitutions at the 3- and 4- positions of the ring, while the 2-substituent contains a thio-methyl spacer and a polar group (i.e. amide or 2-imidazole) (Rosentreter et al. 2001; Rosentreter et al. 2003). Biological evaluation of these compounds has been reported as functional assays at A_2B_AR expressed in Chinese Hamster Ovary (CHO) cells and radioligand binding assays at the remaining ARs (Beukers et al. 2004). Among these derivatives, the compound 2-[(6-amino-3,5-dicyano-4-(4-(cyclopropylmethoxy)phenyl)pyridin-2-yl)thio]acetamide (BAY 606583) has been evaluated for the study of the role of the A_2B_AR in modulating the activity of the immune system (van der Hoeven et al. 2011) and for its possible use in case of heart ischemia (Eckle et al. 2007). Table 2 presents some examples of non-nucleoside A_2B_AR agonists. The activity data are taken from a review article by Baraldi and co-workers (Baraldi et al. 2009).

**Table 2:** **Non-nucleoside A**_**2B**_ **AR agonists analysed in this work (see Additional file** **1** **for structural details)**

cpd	A_1_AR***K***_i_, nM	A_2A_AR***K***_i_, nM	A_2B_AR EC_50_, nM	A_3_AR***K***_i_, nM
**7**	2.4	28	19	171
**8**	2.6	28	12	538
**9**	7.0	214	9	24
**10**	4.4	21	10	104
**11**	2.0	105	34	74
**12**	> 10000	> 10000	3	> 10000

The activity data are taken from Baraldi and co-workers (Baraldi et al. 2009).

The aim of this work is to analyse the binding mode of both nucleoside and non-nucleoside A_2B_AR agonists at 3D models of this AR subtype. The compounds presented in Tables 1 and 2 have been considered for this analysis (see Additional file 1 for structural details). As a preliminary step, the study starts from the rebuilding of homology models of the human A_2B_AR by using as templates the crystal structures of the human A_2A_AR that is the member of AR family presenting also the highest sequence conservation with the A_2B_AR. The binding mode of ligands is then simulated by molecular docking tools, followed by energy minimization and post-docking analysis. In this study, it is not possible to depict any correlation between binding scores or interaction energies and activity data, as the potencies of the compounds have been measured as EC_50_ with functional studies and not as *K*_i_ affinity with radioligand binding assays. Furthermore, the study provides an interpretation of the interaction features for both series of ligands at the A_2B_AR but do not consider the interaction with the other AR subtypes. Hence, the results of this study may provide useful data for the design of A_2B_AR agonists but not for the improvement of selectivity versus the other ARs.

## Methods

All molecular modelling studies were performed on a Core i7 CPU (PIV 2.20 GHZ) PC workstation. Homology modelling, energy minimization, and docking studies were carried out using Molecular Operating Environment (MOE, version 2010.10) suite (Molecular Operating Environment). All ligand structures were optimized using RHF/AM1 semiempirical calculations and the software package MOPAC (Stewart 1990) implemented in MOE was used for these calculations.

### Homology modelling of the human A_2B_AR

Homology models of the human A_2B_AR were built using the recently solved X-ray structures of the human A_2A_AR in complex with Ado and UK-432097 as templates, both structures being retrieved from Protein Data Bank (pdb code: 2YDO; 3.0-Å resolution (Lebon et al. 2011) and pdb code: 3QAK; 2.7-Å resolution (Xu et al. 2011), respectively). A multiple alignment of the AR primary sequences was built within MOE as a preliminary step. For all A_2B_AR models, the boundaries identified from the used X-ray crystal structure of A_2A_AR were then applied for the corresponding sequences of the transmembrane (TM) helices of the A_2B_AR. The missing loop domains were built by the loop search method implemented in MOE. Once the heavy atoms were modelled, all hydrogen atoms were added, and the protein coordinates were then minimized with MOE using the AMBER99 force field (Cornell et al. 1995) until the Root Mean Square (RMS) gradient of the potential energy was less than 0.05 kJ mol^-1^ Å^-1^. Reliability and quality of these models were checked using the Protein Geometry Monitor application within MOE, which provides a variety of stereochemical measurements for inspection of the structural quality in a given protein, like backbone bond lengths, angles and dihedrals, Ramachandran φ-ψ dihedral plots, and quality of side chain rotamer and non-bonded contact.

### Molecular docking analysis

All compound structures were docked into the binding site of the two A_2B_AR models using the MOE Dock tool. This method is divided into a number of stages: *Conformational Analysis of ligands*. The algorithm generated conformations from a single 3D conformation by conducting a systematic search. In this way, all combinations of angles were created for each ligand. *Placement*. A collection of poses was generated from the pool of ligand conformations using Triangle Matcher placement method. Poses were generated by superposition of ligand atom triplets and triplet points in the receptor binding site. The receptor site points are alpha sphere centres which represent locations of tight packing. At each iteration a random conformation was selected, a random triplet of ligand atoms and a random triplet of alpha sphere centres were used to determine the pose. *Scoring*. Poses generated by the placement methodology were scored using two available methods implemented in MOE, the *London dG* scoring function which estimates the free energy of binding of the ligand from a given pose, and *Affinity dG* scoring which estimates the enthalpic contribution to the free energy of binding. The top 30 poses for each ligand were output in a MOE database.

### Post docking analysis

The five top-score docking poses of each compound were then subjected to AMBER99 force field energy minimization until the RMS gradient of the potential energy was less than 0.05 kJ mol^-1^ Å^-1^. Receptor residues within 6 Å distance from the ligand were left free to move, while the remaining receptor coordinates were kept fixed. AMBER99 partial charges of receptor and MOPAC output partial charges of ligands were utilized. Once the compound-binding site energy minimization was completed, receptor coordinates were fixed and a second energy minimization stage was performed leaving free to move only compound atoms. MMFF94 force field (Halgren 1996a, b, c, d;Halgren and Nachbar 1996; Halgren 1999a, b) was applied. For each compound, the minimized docking poses were then rescored using *London dG* and *Affinity dG* scoring functions and the *dock-pK*_*i*_ predictor. The latter tool allows the estimation of the p*K*_i_ for each ligand using the “scoring.svl” script retrievable at the SVL exchange service (Chemical Computing Group, Inc. SVL exchange: http://svl.chemcomp.com). The algorithm is based on an empirical scoring function consisting of a directional hydrogen-bonding term, a directional hydrophobic interaction term, and an entropic term (ligand rotatable bonds immobilized in binding). For each compound and at each A_2B_AR model, the top-score docking poses according to at least two out of three scoring functions were selected for final ligand-target interaction analysis.

The interactions between the ligands and the receptors binding site were analysed by using the *IF-E 6.0* tool (Shadnia et al. 2009) retrievable at the SVL exchange service. The program calculates and displays the atomic and residue interaction forces as 3D vectors. It also calculates the per-residue interaction energies, where negative and positive energy values (expressed as kcal mol^-1^) are associated to favourable and unfavourable interactions, respectively. A shell of residues contained within a 10 Å distance from ligand were considered for this analysis.

## Results and discussion

**Figure 2:**
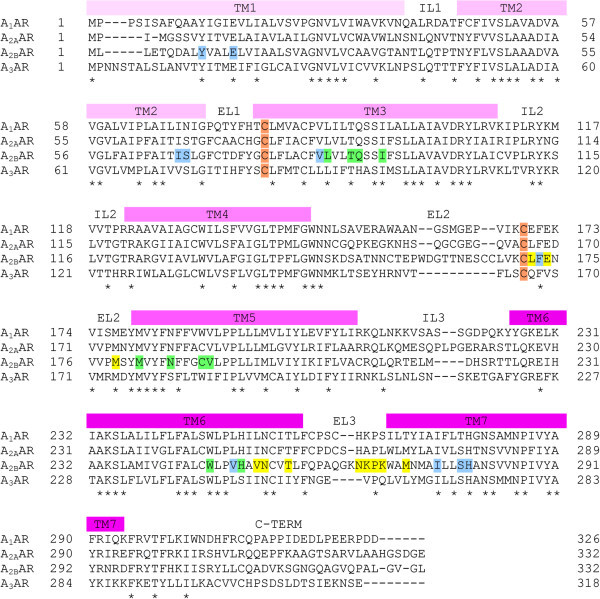
**Sequence alignment of the four human AR subtypes.** Transmembrane (TM), intracellular loop (IL), extracellular loop (EL), and C-terminal (C-TERM) domains are indicated; * symbols indicate sequence identity in all the four subtypes; C letters indicate cysteine residues involved in the disulfide bridge conserved among the four AR subtypes; letters coloured in yellow, cyan, and green indicate the A_2B_AR binding site residues involved in ligand interaction (the colour indexing refers to the binding site subdivision described in Figure 5, see its legend for details).

To simulate the binding mode of nucleoside and non-nucleoside agonists at A_2B_AR and to compare the key ligand-target interaction features of the two structural families of compounds, a molecular docking analysis was performed at homology models of the human A_2B_AR developed by using two recently published crystal structures of the agonist-bound A_2A_AR as templates (pdb code: 2YDO; 3.0-Å resolution (Lebon et al. 2011) and pdb code: 3QAK; 2.7-Å resolution (Xu et al. 2011), in complex with Ado and UK-432097, respectively). The availability of crystal structure the A_2A_AR allows to improve the accuracy of AR homology models, due to the high residue conservation in the primary sequences of the AR subtypes (see Figure 2), sharing a sequence identity of ~57% within the TM domains (Dal Ben et al. 2010b). The residues located within the seven TM domains in the upper part of ARs, corresponding to the ligand binding site, present a conservation (identity) at about 71% (Costanzi et al. 2007). The obtained A_2B_AR homology models were checked by using the Protein Geometry Monitor application within MOE (Environment), which provides a variety of stereochemical measurements for inspection of the structural quality in a given protein, such as backbone bond lengths, angles and dihedrals, Ramachandran φ-ψ dihedral plots, and quality of side chain rotamer and non-bonded contact. The final A_2B_AR models contain a disulfide bridge given by two cysteine residues belonging to TM3 and extracellular loop (EL) 2 domains (Cys78^3.25^ - where 3.25 indicates the residue position within helix (Ballesteros and Weinstein 1995) - and Cys171, respectively), in agreement with recent mutagenesis studies (Schiedel et al. 2011) and as observed in the case of recently reported modelling analyses on this AR subtype (Sherbiny et al. 2009; Thimm et al. 2013; Inamdar et al. 2013).

The A_2B_AR structures were then used as target for the docking analysis of synthesised derivatives. All ligand structures were optimized using RHF/AM1 semi-empirical calculations and the software package MOPAC implemented in MOE was utilized for these calculations (Stewart 1990). The compounds were then docked into the binding site of the A_2B_AR models by using the MOE Dock tool. Top-score docking poses of each compound were subjected to energy minimization; in this phase the binding site residues within 6 Å proximity were left free to move and to adapt their conformation to the ligand moiety. Once this step was completed, a second minimization phase was performed keeping fixed the receptor coordinates. The obtained ligand-target complexes were then rescored using three available methods implemented in MOE: the *London dG* scoring function that estimates the free energy of binding of the ligand from a given pose; the *Affinity dG* scoring tool that estimates the enthalpic contribution to the free energy of binding; the *dock-pK*_*i*_ predictor that uses the MOE *scoring.svl* script to estimate for each ligand a p*K*_i_ value, which is described by the H-bonds, transition metal interactions, and hydrophobic interactions energy. For each compound, the top-score docking pose at each A_2B_AR model, according to at least two out of three scoring functions, was selected for final ligand-target interaction analysis.

The binding sites of the two developed A_2B_AR models are very similar considering both receptor residues orientation and pocket volumes. Slight differences are still observable, due to diverse arrangements of some residues detectable even at the two A_2A_AR crystal structures templates. For example, a glutamate residue (Glu169 in the A_2A_AR) located within EL2 segment makes a clear H-bond interaction with the *N*^6^-amino group of Ado in the 2YDO crystal structure, while the same residue is oriented in opposite direction when observed within the 3QAK X-ray. This residue presents different orientation even considering four previously reported crystal structures of the A_2A_AR in complex with ZM241385 antagonist (Jaakola et al. 2008; Dore et al. 2011; Hino et al. 2012; Liu et al. 2012). Furthermore, a recent report on mutagenesis studies at the A_2A_AR shows that the mutation of this residue does not significantly modify the potency of nucleoside and non-nucleoside agonists at this AR subtype (Lane et al. 2012). These data suggest that the interaction with this residue is important but maybe not critical for the ligand binding to the receptor. Analogously to what observed at the 2YDO and 3QAK crystal structures, comparable arrangements are respectively observed for the corresponding residue (Glu174) at the two A_2B_AR models. In the second case, this glutamate points towards the side chain of Lys269 (EL3) making a strong polar interaction with this amino acid. In any case, most of differences of binding site residue arrangements are observed at EL domains in peripheral regions of binding site and hence have a marginal impact on the binding site size and chemical-physical properties. As consequence, it is not a surprise that the docking analysis of the synthesised compounds at the two receptor models leads to analogue results.

**Figure 3:**
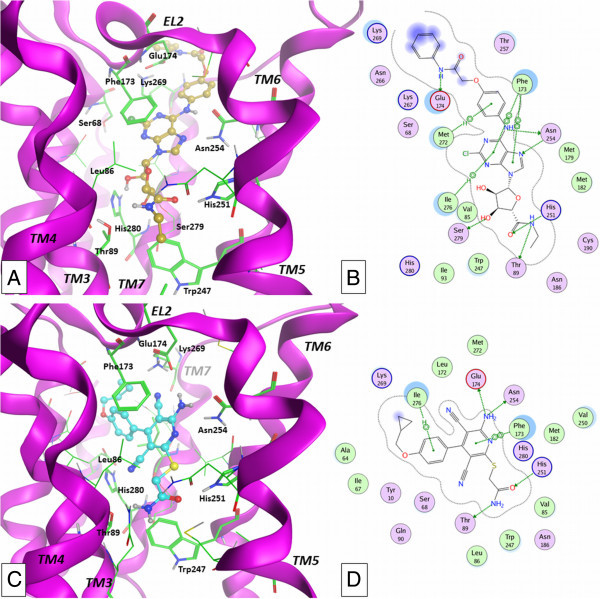
**Results of docking studies.** Docking conformations of nucleoside (compound 5: **A**-**B**; TM4 domain is partially hidden) and non-nucleoside (compound 12: **C**-**D**) derivatives at the 2YDO-based A_2B_AR model and ligand-target interaction plots as computed by MOE software.

Considering the nucleoside agonists, the docking conformations share a common motif, presenting the Ado/NECA derivatives located in the binding site similarly to the co-crystallized nucleoside agonists. In detail, adenine scaffold is positioned between TM3, TM6, and TM7, with the 8- and 9-positions pointing towards the core of the receptor, while the 2- and *N*^6^-substituents are externally located (Figure 3A-B). The adenine plane is stabilized by an aromatic stacking interaction with the AR conserved phenylalanine residue in EL2 domain (Phe173 in A_2B_AR), while the *N*^6^-amino group and the N7 atom interact through H-bonding with a conserved asparagine (Asn254^6.55^ in A_2B_AR). The *N*^6^-amino group makes also H-bonding with Glu174 in the case of the 2YDO-based A_2B_AR model but not in the case of the 3QAK-based model, due to the different orientation of the amino acid side chain. Considering again the 2YDO-based A_2B_AR model, in the case of the presence of substituents at the *N*^6^-position of ligands (compounds 3–5), a rearrangement of Glu174 side chain is observed during post-docking minimization stage. The obtained conformation of the amino acid is comparable to the one of the corresponding residue in the 3QAK-based model and is able to make a polar interaction with Lys269. The *N*^6^-substituents contain polar groups able to give polar interactions with residues in their proximity. In particular, compounds 3 and 4 contain a substituted carbonyl-hydrazine at the 6-position, with the two polar hydrogens of hydrazine group oppositely oriented and pointing towards Asn254^6.55^ and Glu174 polar oxygens. In the case of compound 5, the phenyl ring directly bound to the *N*^6^-amine group causes a major rearrangement of Glu174 side chain (2YDO-based A_2B_AR model) during post-docking minimization step, leading to a conformation highly similar to the same residue in 3QAK-based model. The obtained minimized systems present in both cases the Glu174- Lys269 polar interaction, with an additional H-bonding between Glu174 and the polar hydrogen atom of amide function within the *N*^6^-substituent. Further polar interaction is possible between the carbonyl group of the same amide function of 5 and the side chain of Asn266 (EL3). The 2-substituent (compounds 1, 2, 4–6) is located between TM1, TM2, TM7, and EL2 with the position of phenyl ring (compounds 1, 2, 6) roughly corresponding to the one occupied by the urea group within the 2-substituent of co-crystallized UK-432097 agonist. The ribose moiety is located into the TM helices bundle, in a region between TM2, TM3, TM6, and TM7 in close proximity to the conserved tryptophan (Trp247^6.48^ in A_2B_AR) side chain. The ribose ring presents the hydroxyl groups at the 2′- and 3′-position giving H-bond interaction with A_2B_AR His280^7.43^ and Ser279^7.42^ side chains, respectively. The Ado derivatives (1–2) present in 4′-position a hydroxymethyl group located between TM3 and TM6 and interacting with His251^6.52^ side chain, while the 4′-ethylcarboxamido substituent of NECA derivatives (3–6) is located in analogue position and gives H-bond interaction with Thr89^3.36^ and His251^6.52^.

Considering the non-nucleoside pyridine derivatives, the lowest score and most populated family of docking conformations shows the pyridine ring located in rough correspondence to the adenine scaffold of nucleoside agonists and forming a π-stacking interaction with Phe173 (EL2, Figure 3C-D). Considering the positioning of the different substituents within the binding cavity, the orientation of the pyridine agonists results somehow comparable to the one obtained and schematically described by Sherbiny and colleagues (Sherbiny et al. 2009) but significantly different respect to the ones obtained in more recent docking studies at the same AR subtype (Thimm et al. 2013) and at the A_2A_AR (Lane et al. 2012). In detail, the interaction of the scaffold with the A_2B_AR binding site is given as H-bonding between the N1 atom and the 6-amino group of pyridines and the amine and carbonyl groups of Asn254^6.55^ amide function, respectively. This double polar interaction is clearly present only for some derivatives, due to the nature of the 4-substituent that slightly modifies in some cases the orientation of the scaffold. Analogously to the nucleoside agonists, the amino group at the 6-position of pyridines gives a H-bond interaction with Glu174 in the case of the 2YDO-based A_2B_AR model, while in the case of the 3QAK-based model the side chain of the same residue in some cases is not close enough to the ligand amino group and hence not able to provide a clear H-bonding. The thiomethylimidazole (7–11) and thioacetamide (12) groups in 2-position are inserted between TM3, TM5, and TM6 residues. In the case of compounds 7–11, the H-bond donor function of imidazole is oriented towards the oxygen atom of Thr89^3.36^, while the acceptor feature points towards the polar hydrogen of His251^6.52^. In the case of compound 12, analogue interactions are given by the amine and carbonyl functions of thioacetamide group, respectively. The 3-cyano group is inserted in a sub-cavity between Val85^3.32^, Leu86^3.33^, Thr89^3.36^, Ser279^7.42^, and His280^7.43^. No clear interaction with binding site is given by this group, even though the presence of some space between this function and the polar groups of Ser279^7.42^ and His280^7.43^ could allow the presence of a water molecule providing a sort of “bridge-interaction” between ligand and binding site residues, as observed, for example, in the case of a crystal structure of A_2A_AR in complex with ZM241385 (Jaakola et al. 2008). The substituted aromatic group at the 4-position is located in a sub-cavity given by TM1, TM2, TM3, and TM7 residues, in close proximity to Tyr10^1.35^, Ala64^2.61^, Ile67^2.64^, Ser68^2.65^, and Ile276^7.39^. Further residues in proximity of this group are Val85^3.32^ and Phe173. The interaction is mainly hydrophobic, even if polar interaction could be given by the presence of a hydroxyl function on the aromatic substituent (i.e. compounds 8 and 10). Finally, the 5-cyano group points towards the extracellular environment and is located in proximity to the couple of residues Glu174-Lys269.

**Figure 4:**
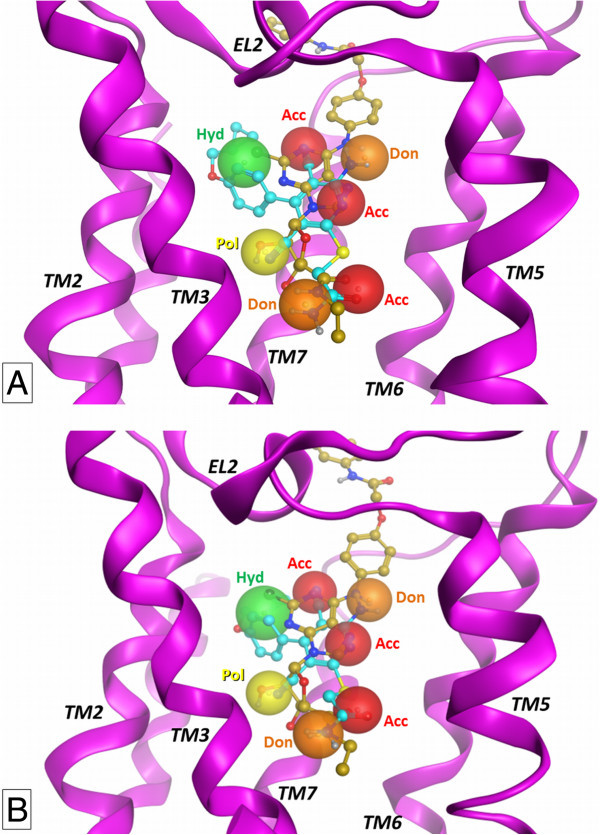
**Comparison of docking conformations.** Superimposition of docking conformations of nucleoside (compound 5) and non-nucleoside (compound 12) derivatives at A_2B_AR at the 2YDO- and 3QAK-based A_2B_AR models (**A** and **B**, respectively). TM4 domain is partially hidden. The matching between functional groups presenting analogue properties among the two molecules are represented as pharmacophoric features (see text for details).

To evaluate the possible common features for the interaction with receptor binding site, the docking conformations of nucleoside and non-nucleoside agonists have been subjected to a comparative analysis performed and here presented considering two different points of views. Firstly, a direct comparison can be made by the superimposition of the representative docking conformations of one nucleoside and one non-nucleoside agonist, with the aim of obtaining, if present, a sort of pharmacophore description. In this sense, Figure 4 shows the superimposition of the docking conformations of compounds 5 and 12 (the most potent compounds from the two ligand families) at the two A_2B_AR models. The results of the superimposition are almost identical at the two receptor models and highlight the presence of a series of common interaction points that can be converted to pharmacophoric features. In details, the 6-amino group of nucleosides and pyridines are located in a similar position, providing a H-bond donor feature able to interact with the H-bond acceptor features given by the carbonyl group of Asn254^6.55^ and, in some cases, by the carboxyl group of Glu174 (EL2). Similarly, the N7 and N1 atoms of nucleosides and pyridines, respectively, are almost superimposed and represent a H-bond acceptor feature located in close proximity with the donor function given by the amine group belonging to amide function of Asn254^6.55^. Furthermore, the 4′-*N*-ethylcarboxamide function of the nucleoside derivative 5 presents the amide carbonyl group and polar hydrogen in a nearly identical position to the analogue functions of 2-thioacetamide group of compound 12, suggesting the importance of the presence in the ligand of a combination of H-bond acceptor and donor features located in that sub-region of the binding site. The carbonyl groups are located in close proximity to a polar hydrogen of His251^6.52^, while the polar hydrogens of the ligand amide groups are located near the polar oxygen of Thr89^3.36^. While the pyridine derivatives present an amide function (compound 12) or an imidazole ring (7–11) both able to provide the two pharmacophoric features, in the case of nucleoside derivatives the two functions can be present only in the case of NECA derivatives (3–6), while the Ado analogues (1, 2) may fit only one of the two polar features. This data is observable already from the 2YDO and 3QAK crystal structures and could in any case explain the general behaviour at ARs according to which the Ado derivatives are generally less potent respect to the corresponding NECA or MECA (4′-*N*-methylcarboxamidoAdo) analogues. The docking position of the 3-cyano group of pyridines roughly corresponds to the position of the nucleosides 2′-hydroxyl function. As reported above, the cyano group is inserted in a sub-cavity between Val85^3.32^, Leu86^3.33^, Thr89^3.36^, Ser279^7.42^, and His280^7.43^, with the presence of some space between this function and the polar groups of Ser279^7.42^ and His280^7.43^. In this sense, the possible presence of a water molecule providing a “bridge-interaction” between ligand and binding site residues could suggest the introduction of a sort of “polar group feature” corresponding to the presence of the 2′-hydroxyl function of nucleosides and the cyano group of pyridines. This polar group could interact with Ser279^7.42^ and His280^7.43^ residues directly (like in the case of nucleosides) or with the aid of a bridging water molecule (in the case of pyridines). The presence of a hydrophobic group at the 2-position of nucleosides and at the 4-position of pyridines suggests the presence of a large hydrophobic feature located within TM1, TM2, and TM7 domains. This group seems critical in particular for pyridine derivatives, as only some nucleoside agonists present a 2-hydrophobic substituent. Finally, the position of the N1 atom of nucleosides is almost coincident to the one of the nitrogen atom of the 5-cyano group of pyridines, suggesting a possible H-bond acceptor function in that position. However, it must be noted that these atoms are oriented towards the extracellular environment and the H-bond donor counterpart could be provided only by a special arrangement of Lys269 (EL3) side chain. We underline that the use of a pharmacophore model in this context is aimed only at helping for the description of an interaction pattern that appears conserved between nucleoside and non-nucleoside agonists. For a development of a proper pharmacophore model, a larger set of molecules and *K*_i_ affinity data should be employed.

**Figure 5:**
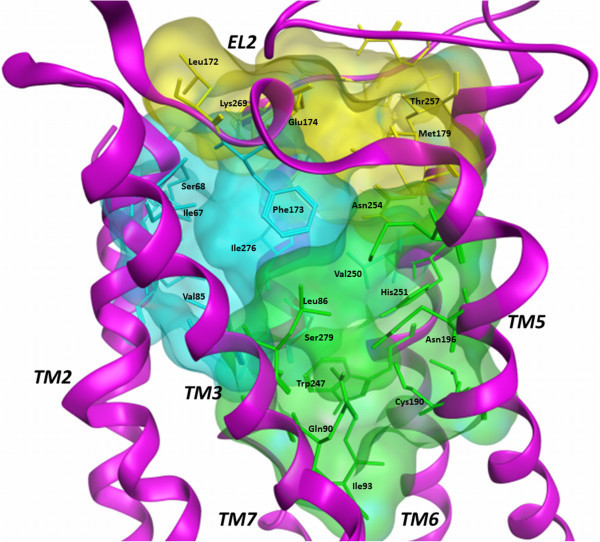
**Analysis of the role of binding site residues for the interaction with ligands.** The binding site is divided in three sections representing the TM1-TM2-TM7 and the TM3-TM5-TM6 regions of the cavity (cyan and green, respectively) and the entrance of the binding site (yellow). TM4 domain is partially hidden.

**Figure 6:**
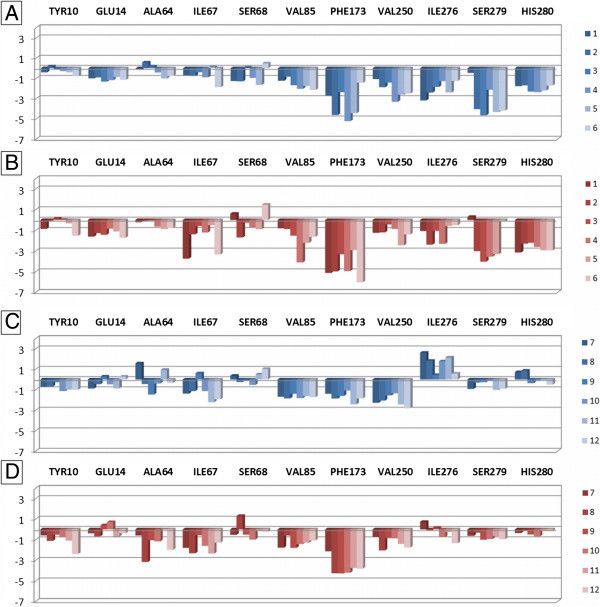
**Plot of interaction energies for residues belonging to the section 1 of the A**_**2B**_ **AR binding site, calculated with the MOE** ***IF-E 6.0*** **tool.** Data are represented as kcal mol^-1^. The blue and red versions of the plots represent the results obtained at the 2YDO- and 3QAK-A_2B_AR models, respectively. Plots **A** and **B** are referred to the interaction energies of nucleoside derivatives 1–6, while plots **C** and **D** are referred to the non-nucleoside derivatives 7–12.

A second approach to compare the binding modes of the different families of compounds at A_2B_AR consists in evaluating the interaction of ligands with each binding site residue, obtaining a set of data representing the different effect of the A_2B_AR residues during the ligand-target interaction. This analysis has been performed by the use of the *IF-E 6.0* (Shadnia et al. 2009) tool retrievable at the SVL exchange service. This method was already used by our research group for the analysis of ligand-target interaction for a series of potent A_3_AR agonists (Dal Ben et al. 2010a). The script calculates and displays atomic and residue interaction forces as 3D vectors. It also calculates the per-residue interaction energies (values in kcal mol^-1^), where negative and positive energy values are associated to favourable and unfavourable interactions, respectively. The script has been applied to each compound at both A_2B_AR models. To display the results, the binding site residues are divided in three sections (Figure 5). The section 1 contains residues belonging to TM1 (Tyr10^1.35^ and Glu14^1.39^), TM2 (Ala64^2.61^, Ile67^2.64^, and Ser68^2.65^), and TM7 (Ile276^7.39^, Ser279^7.42^, and His280^7.43^) domains, with the insertion also of Val85^3.32^, Phe173 (EL2), and Val250^6.51^. These residues represent the receptor region interacting with ligand adenine and pyridine scaffolds and with the substituents at the 2-position of nucleosides and at the 3- and 4-positions of pyridines. Residues interacting with part of ribose atoms of nucleoside derivatives are also included in this section. Figure 6 represents the obtained results for section 1. The blue and red versions of the plots represent the results obtained at the 2YDO- and 3QAK-A_2B_AR models, respectively. In agreement with what appears from the superimposition of docking conformations (see above), the effect of these residues is different from nucleoside to non-nucleoside ligands as in the first case the 2-substituent of nucleosides is present only in some cases and presents different structural and chemical profiles, while in the second case the 4-substituent of pyridines is large and forming various interactions with binding site residues. In detail, considering the nucleoside derivatives 1–6, it clearly appears the effect of Phe173, Ser279^7.42^, and His280^7.43^ in stabilizing the ligand-target interaction. In particular, Phe173 is the key residue for stabilizing the scaffold position within the binding site through a π-stacking interaction, while Ser279^7.42^ and His280^7.43^ are involved in polar interaction with 2′- and 3′-hydroxyl groups of nucleosides. The further contribution of Val250^6.51^ and Ile276^7.39^ for scaffold stabilization is more evident at 2YDO-A_2B_AR model, while the role of the carbonyl group of Ile67^2.64^ backbone atoms in forming H-bond interaction with the hydroxyl group of 2-substituent of 1 and 6 is clearly evident at 3QAK-based model. The latter residue forms also hydrophobic interaction with the alkynyl group of the nucleosides 1 and 6 (3QAK-based model). Considering the non-nucleoside derivatives 7–12, results highlight the role of Val85^3.32^, Phe173, and Val250^6.51^ in stabilizing the ligand scaffold, while the interaction with Ser279^7.42^ and His280^7.43^ is lacking (see above). Tyr10^1.35^ interacts with compound 12 in 3QAK-based model. Ile67^2.64^ forms hydrophobic interaction with the 4-aromatic group of pyridines, while Ala64^2.61^ forms on the one hand a stable H-bond interaction with the *para*-hydroxyl group of the 4-aromatic substituent of compound 8 (3QAK-based model), on the other hand a hydrophobic interaction with 4-aromatic group of 12. Comparing the results for this section with previously reported data, it can be underlined that Tyr10^1.35^ showed to be critical for compound 12 activity as evidenced from mutagenesis results (Thimm et al. 2013), while Ile67^2.64^ and Ala64^2.61^ were already reported to possibly interact with the 4-substituent of pyridine derivatives (Sherbiny et al. 2009). The effect of Ser279^7.42^ and His280^7.43^ is evident for nucleoside agonists but not so well defined for non-nucleoside derivatives. In particular, the mutation of Ser279^7.42^ to alanine takes to a loss of activity of nucleosides and, on the contrary, to an improvement of non-nucleoside derivative EC_50_ data (Thimm et al. 2013). These results are confirmed at the A_2A_AR (Lane et al. 2012). Our study confirms the effect on nucleosides but does not highlight a direct interaction with non-nucleoside derivatives. The histidine residue in position 7.43 of ARs (His280^7.43^ in A_2B_AR) is reported to be essential for receptor expression and function (Dal Ben et al. 2010b; Thimm et al. 2013). Considering the interaction of this residue in A_2B_AR with ligands, it has been recently reported a molecular modelling study consisting in the rebuilding of the three AR models other than A_2A_AR and the use of the four AR 3D structures for a molecular docking study of antagonists followed by molecular dynamics analysis (Inamdar et al. 2013). Considering the results at the A_2B_AR, this study suggests a possible direct contact of His280^7.43^ with a potent thiazole antagonist. Further recent studies report that the mutation of this histidine leads to a loss of interaction with ligands (Thimm et al. 2013). As reported above, our modelling study does not highlight a direct contact of His280^7.43^ with non-nucleoside derivatives even if we cannot exclude that an interaction of these ligands with this residue and Ser279^7.42^ could be mediated by a water molecule.

**Figure 7:**
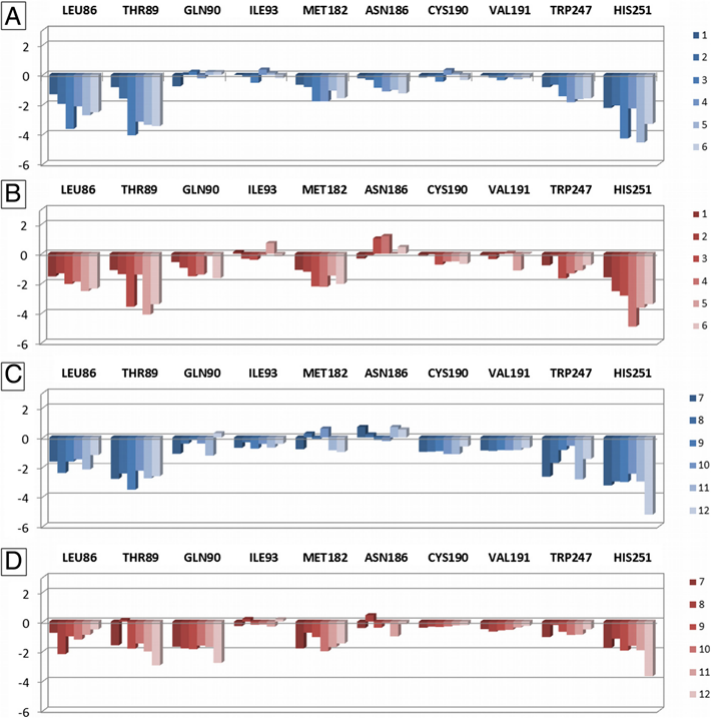
**Plot of interaction energies for residues belonging to the section 2 of the A**_**2B**_ **AR binding site, calculated with the MOE** ***IF-E 6.0*** **tool.** Data are represented as kcal mol^-1^. The blue and red versions of the plots represent the results obtained at the 2YDO- and 3QAK-A_2B_AR models, respectively. Plots **A** and **B** are referred to the interaction energies of nucleoside derivatives 1–6, while plots **C** and **D** are referred to the non-nucleoside derivatives 7–12.

The section 2 contains residues belonging to TM3 (Leu86^3.33^, Thr89^3.36^, Gln90^3.37^, and Ile93^3.40^), TM5 (Met182^5.38^, Asn186^5.42^, Cys190^5.46^, and Val191^5.47^), and TM6 (Trp247^6.48^ and His251^6.52^). These residues are involved in interaction with ribose group of nucleoside analogues (in particular the 4′-position) and with 2-substituent of non-nucleoside pyridine derivatives. Figure 7 represents the obtained results for section 2, again with blue and red versions corresponding to the 2YDO- and 3QAK-A_2B_AR models, respectively. The effect of the receptor residues for the interaction with ligands is comparable considering both nucleoside and non-nucleoside agonists. Firstly, Leu86^3.33^ and Met182^5.38^ form a hydrophobic interaction with the ribose group of nucleosides and with the 2-substituent of pyridines. Secondly, it appears evident the contribution of Thr89^3.36^ and His251^6.52^, in accordance with the results of superimposition experiments (see above). In particular, Thr89^3.36^ provide a stabilizing effect for the polar hydrogen of NECAs 5′-amide group from 3–6, of thiomethylimidazole from 7–11, and of thioacetamide from 12. The effect of this residue is largely reduced in the case of the two Ado derivatives 1–2 that do not present a combination of H-bond acceptor and donor features at the 5′-position. His251^6.52^ cooperates with Thr89^3.36^ in providing a stabilizing effect for ligands. In the case of non-nucleoside derivatives, it is interesting to notate the greater effect of this residue for 12 respect to 7–11 at both A_2B_AR models, suggesting a better interaction of His251^6.52^ for the amide function respect to an imidazole ring. The effect of the other residues of this section is not significant, with the exception in some cases of Gln90^3.37^ (polar interaction with non-nucleoside derivatives in 3QAK-A_2B_AR model) and Trp247^6.48^. In a previous study (Sherbiny et al. 2009), Thr89^3.36^ was suggested as interacting with 3-cyano substituent of 12 while His251^6.52^, Gln90^3.37^, and Asn186^5.42^ were found as interacting (or in proximity) with the amide group of the 2-substituent of the same compound. Asn186^5.42^ appears as not directly interacting with the compounds of this study. On the other hand, a mutagenesis study has evidenced that the mutation of this residue to alanine leads to an increase of potency of both nucleoside and non-nucleoside derivatives (Thimm et al. 2013). The interaction between 3.37 and 5.42 residues is absent in the antagonist-bound A_2A_AR crystal structures but present in the agonist-bound 3QAK X-ray data (in the Ado- and NECA-bound A_2A_AR crystal structures the Gln89^3.37^ is mutated to alanine), hence these residues could have a mechanistic role for the receptor function and could be not necessarily interaction points for ligands (Dal Ben et al. 2013).

**Figure 8:**
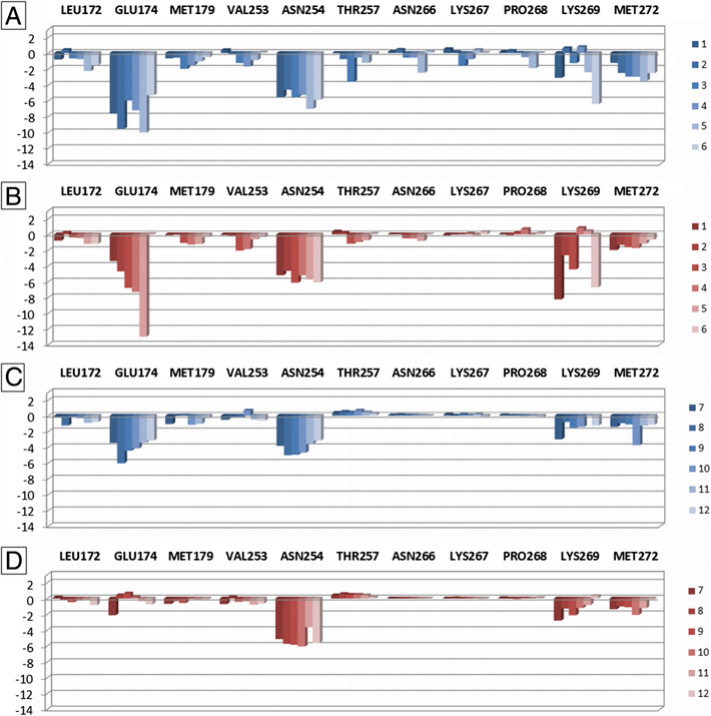
**Plot of interaction energies for residues belonging to the section 3 of the A**_**2B**_ **AR binding site, calculated with the MOE** ***IF-E 6.0*** **tool.** Data are represented as kcal mol^-1^. The blue and red versions of the plots represent the results obtained at the 2YDO- and 3QAK-A_2B_AR models, respectively. Plots **A** and **B** are referred to the interaction energies of nucleoside derivatives 1–6, while plots **C** and **D** are referred to the non-nucleoside derivatives 7–12.

The section 3 contains residues located close or at the entrance of the binding site and belonging to EL2 (Leu172, Glu174, and Met179), TM6 (Val253^6.54^, Asn254^6.55^, and Thr257^6.58^), EL3 (Asn266, Lys267, Pro268, and Lys269), and TM7 (Met272^7.35^). These residues are involved in interaction with the purine and the pyridine scaffold, the C2– and *N*^6^-substituents of nucleosides, and the 4-aromatic substituent, the 5-cyano group, and the 6-amino function of pyridines. Even in this case, the representation is divided in plots for nucleoside and non-nucleoside derivatives at the two 2YDO- and 3QAK-A_2B_AR models (Figure 8). The results confirm the key role of Asn254^6.55^ for the interaction with both families of ligands at both receptor models. The importance of this residue for the interaction of A_2B_AR with ligands has been highlighted even in recently reported studies (Sherbiny et al. 2009; Inamdar et al. 2013). Furthermore, it appears a relevant effect of Glu174 for the interaction with nucleoside derivatives 1–6. The carboxyl function of this residue can interact with the unsubstituted 6-amino group (1, 2, 6) or with polar hydrogens present within the *N*^6^-substituent (3–5). In the case of pyridine derivatives, the effect of this residue is influenced by its conformation, as in the 2YDO-based A_2B_AR model its carboxyl group points towards the 6-amino function of ligands providing H-bond interaction, while in the case of 3QAK-based model the side chain is oriented towards the extracellular environment in proximity of Lys269. Analogue consideration can be made for Lys269, whose side chain points towards Glu174 and the *N*^6^-substituent of nucleoside derivatives. In the case of compounds 1 and 6, the charged amino group of this lysine interacts also with the hydroxyl group within the 2-substituent. In the case of the non-nucleoside derivatives, the Lys269 charged amino group is located in proximity of 5-cyano function without forming interaction with this group. The effect of the remaining amino acids in this section seems not significant.

## Conclusions

In conclusion, this study was aimed at simulating the binding modes of nucleoside and non-nucleoside agonists at two A_2B_AR homology models developed starting from the X-ray structures of the A_2A_AR in complex with Ado and UK-432097 as templates (pdb code: 2YDO and 3QAK, respectively). The docking conformations of nucleoside derivatives were quite expected, while the non-nucleoside derivatives demonstrated to bind to these receptor models in a different way respect to previously reported studies at AR models. The generated and minimized docking conformations were compared by superimposition and by analysis of the interaction with the binding site residues located in ligand proximity. Results showed that, beside the evident structural differences among the two ligand families, the nucleoside and non-nucleoside derivatives bind to the A_2B_AR through a series of conserved interaction points, suggesting a sort of interaction pattern. The findings of this analysis are in agreement with the results of the evaluation of the role of binding site residues for the ligand-target interaction and the conclusions of these studies are in good agreement with mutagenesis and molecular modelling studies reported in the last years. Taken together, these data could be helpful for the design of A_2B_AR agonists belonging to nucleoside or non-nucleoside structural families.

## Authors’ information

DDB, MB, and CL are Assistant Professors of Medicinal Chemistry with interest in molecular modelling and compound synthesis. AT is post-doctoral researcher working in compound synthesis, while RV is Full Professor of Medicinal Chemistry. All authors work at the School of Pharmacy of the University of Camerino, Italy.

## Electronic supplementary material

Additional file 1: Table S1: Nucleoside A2BAR agonists analysed in this work. The activity data are taken from Baraldi and co-workers (Baraldi et al. 2009). **Table S2.** Non-nucleoside A2BAR agonists analysed in this work. The activity data are taken from Baraldi and co-workers (Baraldi et al. 2009). (PDF 48 KB)

## Abbreviations

Ado: Adenosine
AR: Adenosine receptor
GPCR: G Protein-coupled receptor
CHO: Chinese hamster ovary
MOE: Molecular operating environment
pdb: protein data bank
TM: Transmembrane
RMS: Root mean square
EL: Extracellular loop.
