# Supplementary material for: Simulation and comparative analysis of binding modes of nucleoside and non-nucleoside agonists at the A2B adenosine receptor
Source: In Silico Pharmacol. 2013 Dec 20;1:24. doi: 10.1186/2193-9616-1-24 (PMC4215817; doi:10.1186/2193-9616-1-24)
Supplement: Supplementary file 1 — Additional file 1: Table S1: Nucleoside A2BAR agonists analysed in this work. The activity data are taken from Baraldi and co-workers (Baraldi et al. 2009). Table S2. Non-nucleoside A2BAR agonists analysed in this work. The activity data are taken from Baraldi and co-workers (Baraldi et al. 2009). (PDF 48 KB) [file 40203_2013_21_MOESM1_ESM.pdf]

Table 1. Nucleoside A_{2B}AR agonists analysed in this work. The activity data are taken from Baraldi and co-workers (Baraldi et al. 2009).

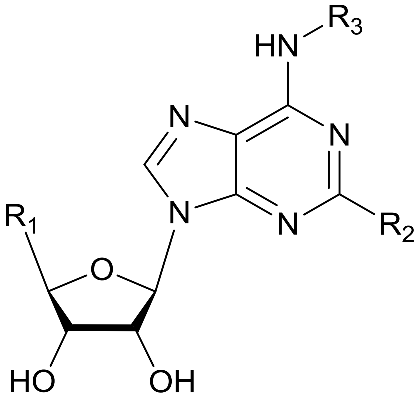

cpd	R_1	R_2	R_3	A_1AR K_i, nM	A_{2A}AR K_i, nM	A_{2B}AR EC_{50}, nM	A_3AR K_i, nM
**1** S-PHPADO	CH_2OH	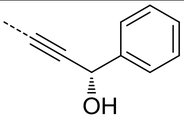	H	0.67	1.8	920	1.4
**2**	CH_2OH	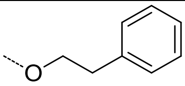	H	221	9.3	3490	54.2
**3**	CONH-Et	H	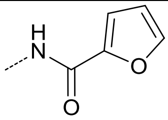	1050	1550	82	> 5000
**4**	CONH-Et	Cl	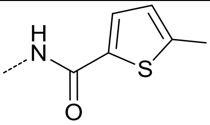	2600	4100	175	> 5000
**5**	CONH-Et	Cl	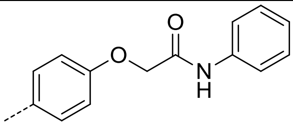	30.5	> 1000	42.6	107
**6** S-PHPNECA	CONH-Et	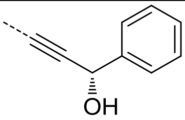	H	2.1	2.0	220	0.75

Table 2. Non-nucleoside $A_{2B}AR$ agonists analysed in this work. The activity data are taken from Baraldi and co-workers (Baraldi et al. 2009).

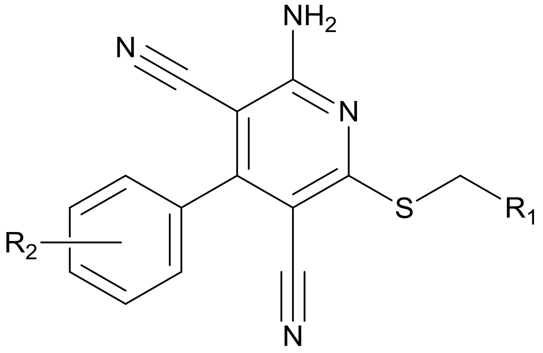

cpd	R_1	R_2	A_1AR K_i, nM	$A_{2A}AR$ K_i, nM	$A_{2B}AR$ EC_{50}, nM	A_3AR K_i, nM
**7** LUF 5833	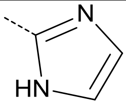	H	2.4	28	19	171
**8** LUF 5834	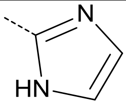	4-OH	2.6	28	12	538
**9** LUF 5845	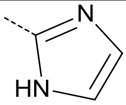	4-OMe	7.0	214	9	24
**10** LUF 5835	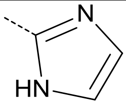	3-OH	4.4	21	10	104
**11** LUF 5844	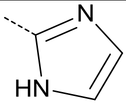	3-OMe	2.0	105	34	74
**12** BAY 606583	$CONH_2$	4-cyclopropylmethoxy	> 10000	> 10000	3	> 10000
